# Investigation of Polymer/Si Thin Film Tandem Solar Cell Using TCAD Numerical Simulation

**DOI:** 10.3390/polym15092049

**Published:** 2023-04-26

**Authors:** Mohamed Okil, Ahmed Shaker, Mostafa M. Salah, Tarek M. Abdolkader, Ibrahim S. Ahmed

**Affiliations:** 1 Department of Basic Engineering Sciences, Benha Faculty of Engineering, Benha University, Benha 13512, Egypt; mohamed.okil@bhit.bu.edu.eg (M.O.); tarek.hassan01@bhit.bu.edu.eg (T.M.A.); ibrahim.maged@bhit.bu.edu.eg (I.S.A.); 2 Faculty of Engineering, Ain Shams University, Cairo 11535, Egypt; 3 Electrical Engineering Department, Future University in Egypt, Cairo 11835, Egypt

**Keywords:** all-thin-film, polymer-based cell, c-Si, Tandem solar cell, VBO, current matching, TCAD simulation

## Abstract

The current study introduces a two-terminal (2T) thin-film tandem solar cell (TSC) comprised of a polymer-based top sub cell and a thin crystalline silicon (c-Si) bottom sub cell. The photoactive layer of the top sub cell is a blend of PDTBTBz-2F as a polymer donor and PC_71_BM as a fullerene acceptor. Initially, a calibration of the two sub cells is carried out against experimental studies, providing a power conversion efficiency (PCE) of 9.88% for the top sub cell and 14.26% for the bottom sub cell. Upon incorporating both sub cells in a polymer/Si TSC, the resulting cell shows a PCE of 20.45% and a short circuit current density (*J*_sc_) of 13.40 mA/cm^2^. Then, we optimize the tandem performance by controlling the valence band offset (VBO) of the polymer top cell. Furthermore, we investigate the impact of varying the top absorber defect density and the thicknesses of both absorber layers in an attempt to obtain the maximum obtainable PCE. After optimizing the tandem cell and at the designed current matching condition, the *J*_sc_ and PCE of the tandem cell are improved to 16.43 mA/cm^2^ and 28.41%, respectively. Based on this TCAD simulation study, a tandem configuration established from an all thin-film model may be feasible for wearable electronics applications. All simulations utilize the Silvaco Atlas package where the cells are subjected to standard one Sun (AM1.5G, 1000 W/m^2^) spectrum illumination.

## 1. Introduction

Photovoltaics propose promising solutions to the energy demand issue. Various types of solar cells have been widely presented and published. The central goal of recent research is to achieve efficient solar cells with a low cost of fabrication [1,2,3]. The generations of solar cells can be summarized in four generations. First, a generation based on the crystalline-silicon (c-Si), and gallium arsenide. Second, a generation based on amorphous and microcrystalline thin film silicon, copper indium gallium selenide (CIGS), cadmium sulfide (CdS), and cadmium telluride (CdTe). New compounds like organics, polymers and perovskites, and structures like tandem solar cells (TSCs) are the base of the third generation [4,5,6,7,8]. According to the non-toxicity, stability, abundancy, and well-known technology of silicon, silicon solar cells demonstrate the highest share of solar cells’ market. The power conversion efficiency (PCE) of silicon solar cells exceeds 26% [9]. Additionally, many efforts have been conducted in order to achieve low-cost silicon solar cells [10,11,12,13,14]. Recently, a generation based on the insertion of the stable inorganic generation into the flexible, and low-cost polymers like metal-nanoparticles, and metal-oxides has been developed.

Bulk heterojunction polymer solar cells are gaining attention as they have the potential to provide flexible, lightweight, and cost-effective alternatives to silicon-based solar cells [15]. Researchers have been working on improving the charge selectivity of inverted polymer-based solar cells by inserting a sub monolayer of dyes containing functional cyano-carboxylic at the interface between the inorganic metal oxide (ZnO) and organic active layer, resulting in an improved conversion efficiency to 3.52% [16]. Y. Yan et al. [17] recently synthesized and used TiO_2_:TOPD as the electron transport layer in single-junction inverted polymer solar cells, resulting in a high fill factor (FF) of 72% and the best power conversion efficiency (PCE) of 10.55%, making it one of the best single-junction inverted polymer solar cells ever reported, with superior stability under ambient conditions when compared to conventional device configuration.

The maximum PCE of single-junction solar cells is limited to 33.7% [18]. This limitation can be explained by the absorption in these cells being limited to the photons with an energy gap higher than or equal the energy gap of the utilized materials. TSCs propose a favorable solution to this limitation. The structure of this type of solar cells contains sub cells with different absorber materials to absorb most of the incident spectrum [19]. The maximum efficiency of a tandem with infinity sub cells is theoretically 68.2% [20]. Tandems can be established from any two or more complementary materials to absorb most of the radiation spectrum where the power spectrum is divided amongst the multiple absorbers. Normally, a two-junction tandem configuration comprises a wide bandgap top sub cell which absorbs the low wavelength photons and a narrow bandgap sub cell to absorb the high wavelength photons. Numerous combinations of sub cells have been researched. These include All-perovskite (meaning perovskite top sub cell and perovskite bottom sub cell) [21,22,23,24], All-organic [25,26,27], All-polymer [28,29], perovskite/silicon [30,31,32], perovskite/CIGS [9,33], and many more.

In order to provide nontoxic, eco-friendly, flexible solar cells, some research studies have been provided. Some of these included CIGS as a bottom sub cell [34,35,36] while others included Silicon [37,38]. Compared to Silicon, it is recognized that material availability is an issue in CIGS solar cells as indium and gallium are rare metals and, consequently, have expensive prices. So, the cost of processing CIGS-based solar cells is higher than that of Si. Furthermore, the CdS buffer layer encountered in CIGS cells usually uses carcinogenic cadmium, which is quite toxic. This issue puts a limit on CIGS fabrication, unlike Si, which has environmentally friendly materials. In addition, polymer solar cells receive extensive attention because they are lightweight and flexible in addition to other third-generation advantages [39]. Thus, the integration of polymer and Silicon in a TSC deserves more attention. Implementing polymer/silicon TSC can achieve higher efficiencies than single-junction solar cells. In addition to reduced material cost compared to other Silicon based TSCs, flexibility and lightweight are other advantages that may make thin film polymer/silicon TSC suitable for a wider range of applications.

This paper proposes a 2T monolithic TSC combining a top polymer-based sub cell with a bottom thin film c-Si sub cell. Solar cells established on these materials, which are cost-effective to manufacture, are considered environmentally sustainable. When all of the layers are thin film, a flexible tandem can be produced and employed in various uses, including wearable devices. We calibrate the separate sub cells against experimental investigations to validate our simulator, considering the proper geometric and physical considerations before modeling our suggested TSC. Next, we optimize the tandem performance by tuning the valence band offset (VBO) of the top sub cell. Then, the influence of varying the top absorber defect density on the tandem performance is investigated. Furthermore, we vary the thickness of both absorbers to boost the tandem efficiency. Finally, the current matching point (CMP) is inspected for maximum obtainable PCE. The provided TCAD simulation study can shed light on an emerging technology that could become one of the top alternative materials for thin Si-based TSCs.

## 2. Simulation Approach and Device Structures

### 2.1. Silvaco Atlas Numerical Approach

Various software packages can be utilized to assess the performance of single or multijunction solar cells, like SCAPS [40,41], Silvaco [6,12,42], COMSOL [43], AMPS [44], and wxAMPS [45]. This study uses the Silvaco TCAD simulator to model and simulate the proposed tandem cell’s electrical and optical characteristics. The numerical simulation discretizes the solution of the semiconductor carriers’ equations through a specified grid [46]. First, an illumined input source is used (AM1.5G). Then, the photogeneration rates are obtained and incorporated into the continuity equation generation terms. In optoelectronic device simulation, photogeneration and optical ray tracing models are computed together at each bias. The real part of the refractive index is used to calculate the optical intensity in the optical ray tracing model. On the other hand, the photogeneration model calculates a new carrier concentration using the extinction coefficient. After that, the required terminal currents are attained using an electrical simulation, and the transport properties are simulated using the drift-diffusion model.

The physical models incorporated into Atlas are selected as follows. The Shockley–Read–Hall (SRH) mechanism allows the consideration of the trap recombination through the bandgap defect levels of the simulated material. In addition, the models for optical recombination, Fermi-Dirac distribution, and concentration-dependent mobility are also utilized. In a 2T TSC, the two sub cells should be interconnected using a tunnel junction or thin interlayers of Silver (Ag) or Gold (Au) [47]. Additionally, an indium oxide (InO) interlayer, which has been experimentally verified to be efficient for both optical and electrical behavior, can be used as an interlayer [48]. In this study, we utilize a lumped resistance as a connecting layer between the two sub cells allowing the current transports across the tandem sub cells [49].

### 2.2. Subcell Configurations and Simulation Parameters

**Figure 1:**
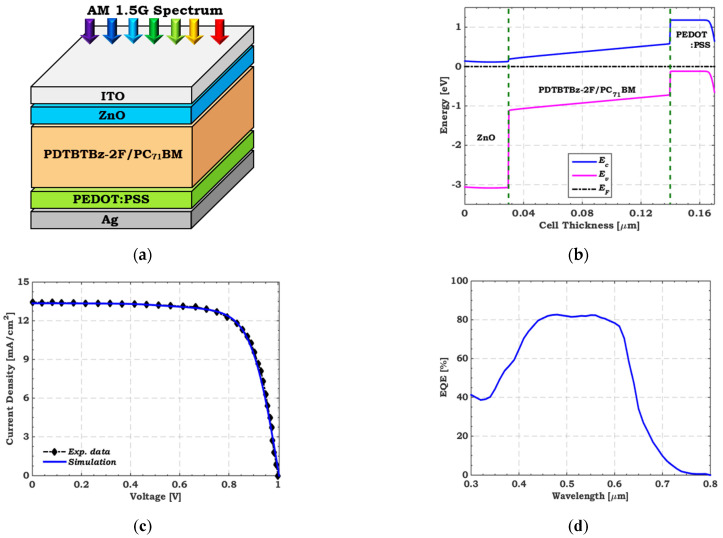
(**a**) Basic layers structure, (**b**) Energy band profile after contact at the dark condition, (**c**) Comparison of the simulated *J–V* curve with the measured data [28] under AM 1.5 illumination condition, and (**d**) *EQE* of a polymer-based cell.

An n–i–p heterojunction polymer top cell is offered on the basis of a practical structure of an experimental solar cell [28]. The active layer blends PDTBTBz-2F as the polymer donor and PC_71_BM as the fullerene acceptor. Photogenerated electron-hole pairs (EHP) are extracted by sandwiching the active layer between low n-type doped Zinc oxide (ZnO) and high-doped p^+^-PEDOT:PSS layers. The schematic structure and energy band profile of the polymer-based top cell are illustrated in Figure 1a,b, respectively. As illustrated in Figure 1b, the band bending implies that the built-in potential emerges on the active layer (i.e., intrinsic regions) in a direction where electrons and holes are separated to the electron transport layer (ETL) and the hole transport layer (HTL), respectively. Table 1 presents the layers’ essential parameters obtained from previously available investigations [28,50,51,52]. Table 2 also includes defect parameters in the absorber layer and at the two interfaces (ZnO/Polymer and Polymer/PEDOT:PSS). The front indium-doped tin oxide (ITO) contact has a work function of 4.4 eV, whereas the work function of the back Ag contact is considered 4.25 eV. The illuminated (AM1.5G) current-voltage (*J–V*) curves are presented in Figure 1c for both simulated and experimental data [28]. In addition, the simulated external quantum efficiency (*EQE*) curve is also shown in Figure 1d. The corresponding simulated photovoltaic (PV) parameters are listed in Table 3, which agree with the experimental results.

**Figure 2:**
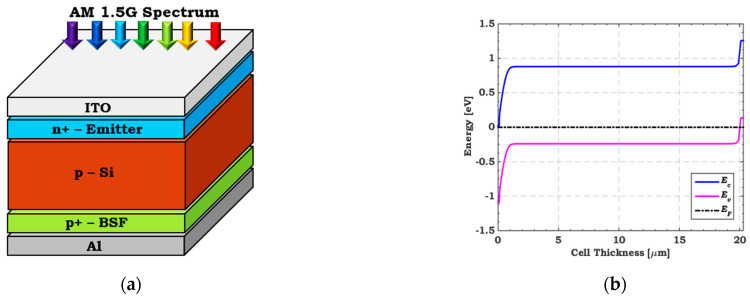

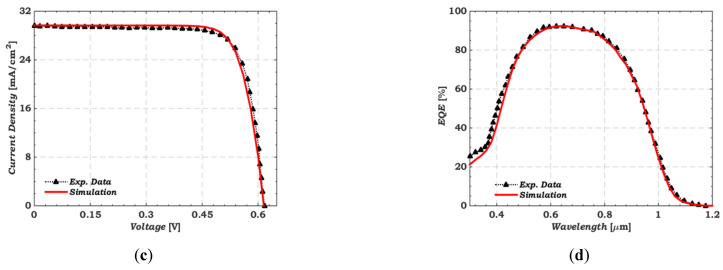
(**a**) Basic layers structure, (**b**) Energy band profile, (**c**) Comparison of the simulated *J–V* characteristics, and (**d**) *EQE* with the measured data [53] of thin-film c-Si cell under AM 1.5 illumination condition.

On the other hand, we calibrate an n^+^–p–p^+^ homojunction c-Si bottom cell against an experimental thin film c-Si cell [53]. The manufacturing processes of the thin Si cell were as follows: (1) a 380 μm Czochralski grade n-type c-Si wafer was etched to create a 20 μm thin c-Si substrate, (2) the spin-on-dopant technique was utilized to produce the back surface field (BSF) and emitter regions, and (3) Al electrodes were defined [53]. The bottom Si cell’s schematic structure and energy band diagram are exhibited in Figure 2a,b, respectively. Table 1 presents the main factors of the layers [54]. Furthermore, the Atlas package includes preset settings for carrier mobility and lifetime. The illuminated *J–V* and *EQE* curves are displayed in Figure 2c,d for both simulated and experimental data [53]. Table 3 lists the PV parameters, demonstrating a close match between the simulated and experimental findings [53]. The agreement for both polymer and Si cells validates the simulation model used within the Silvaco Atlas package.

**Table 1:** Basic parameters of sub cells layers.

Parameters	Description	ZnO	PDTBTBz-2F:PC_71_BM	PEDOT:PSS	n + Si	p Si	p + Si
*t* (μm)	Thickness	0.03	0.11	0.03	0.10	20	0.20
*E_g_* (eV)	Energy gap	3.2	1.3	1.3	1.12	1.12	1.12
*χ* (eV)	Electron affinity	4.26	4.2	3.6	4.05	4.05	4.05
*ε_r_*	Relative permittivity	9	3	3.5	11.7	11.7	11.7
*μ_n_* (cm^2^/Vs)	Electron mobility	200	9 × 10^−4^	1 × 10^−4^	Atlas default values		
*μ_p_* (cm^2^/Vs)	Hole mobility	5	1 × 10^−3^	2 × 10^−5^			
*N_c_* (cm^−3^)	Conduction band effective DOS	2 × 10^18^	1 × 10^20^	1 × 10^21^	2.8×10^19^	2.8 × 10^19^	2.8 × 10^19^
*N_v_* (cm^−3^)	Valence band effective DOS	1.8 × 10^19^	1×10^20^	1 × 10^21^	1 × 10^19^	1×10^19^	1 × 10^19^
*N_D_* (cm^−3^)	Donor concentration	1 × 10^17^	-	-	1 × 10^19^	-	-
*N_A_* (cm^−3^)	Acceptor concentration	-	-	1 × 10^19^	-	1 × 10^15^	1 × 10^20^
*References*		[50]	[7,8,28,51]	[52]	[54]	[54]	[54]

**Table 2:** Defects parameters in the top absorber layer and at the interfaces [7,8].

Parameter	Interface Defects		Bulk Defects
	ZnO/Polymer	Polymer/PEDOT:PSS	Polymer
Defect type	Neutral	Neutral	Acceptor
Carriers capture cross-section	1 × 10^−19^ cm^2^	1 × 10^−19^ cm^2^	1 × 10^−19^ cm^2^
Energetic distribution	Single	Single	Single
Energy level to the highest *E_v_*	0.6 eV	0.6 eV	0.6 eV
Total density	1 × 10^7^ cm^−2^	1 × 10^7^ cm^−2^	1 × 10^11^ cm^−3^

**Table 3:** A comparison between simulated and experimental PV parameters of polymer and thin-film c-Si sub cells.

PV Parameters		*V*_oc_ (V)	*J*_sc_ (mA/cm^2^)	FF (%)	PCE (%)
PDTBTBz-2F: PC_71_BM Cell	Exp. data	0.98 ± 0.02	13.38 ± 0.16	73 ± 1	9.52 ± 0.23
	Simulation	1	13.35	73.70	9.88
c-Si Cell	Exp. data	0.617	29.60	77.90	14.30
	Simulation	0.615	29.66	78.16	14.26

### 2.3. Initial Polymer/Si Tandem Cell

**Figure 3:**
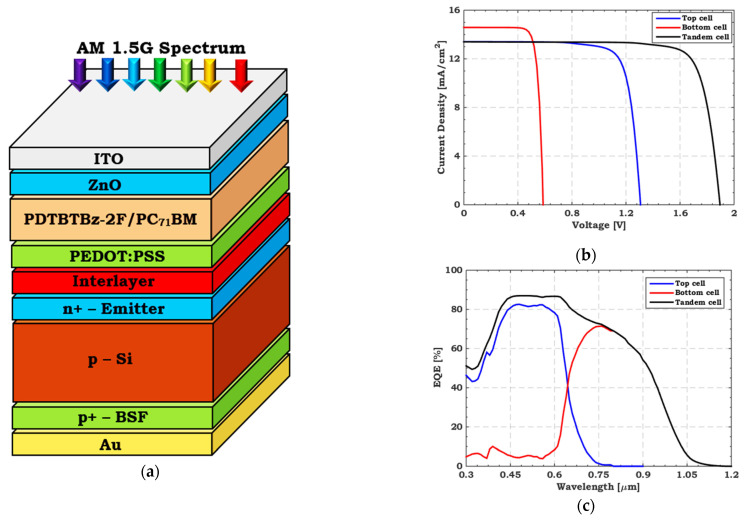
(**a**) Basic layers structure, (**b**) Illuminated *J–V,* and (**c**) *EQE* curves of initial polymer/Si TSC.

This sub section presents the suggested structure of the polymer/Si TSC, as shown in Figure 3a. Here, the tandem sub cells are connected via an interlayer (a very thin metallic film or a transparent conductive oxide) [55,56,57] that performs as a recombination layer that is modeled by a lumped resistance as indicated previously. Furthermore, the lower current that passes through either sub cell controls the TSC current. Once the two sub cells are stacked, the CMP should be maintained to reduce the loss. The designed configuration combines a wide bandgap polymer top cell with a thin c-Si bottom cell. Figure 3b,c display the simulation results (*J–V* and *EQE*) regarding the TSC. The corresponding PV parameters are as follows: *J*_sc_ = 13.40 mA/cm^2^, *V*_oc_ = 1.89 V, FF = 80.54%, and PCE = 20.45%. The results show that the smallest sub cell current regulates the tandem current, while the tandem *V*_oc_ is almost equivalent to the sum of the standalone sub cells’ *V*_oc_.

## 3. Results and Discussions

In this section, we handle the optimization steps for the polymer/Si TSC. First, the VBO impact of the front sub cell on the TSC performance is examined. Then, we investigate the influence of changing the defect density of the top absorber on tandem working metrics. Furthermore, the influence of both absorber thicknesses on tandem performance is investigated. Finally, the CMP is inspected for maximum conversion efficiency.

### 3.1. Valence Band Offset of the Top Cell

Generally, ETLs and HTLs help to extract photogenerated EHP from the absorber layer, which is then transported to both contacts. The selectivity of the carriers’ transport layers influences the performance of solar cells. As a result, perfect transport layers can significantly reduce interfacial recombination. Energetics and transport characteristics like band alignment and charge carrier mobility primarily define solar cell performance. An optimum band offset can be achieved using an appropriate band alignment at both interfaces (ETL/polymer and polymer/HTL) [58]. Furthermore, CBO and VBO play a vital role in determining *V*_oc_ and, thus, cell efficiency, and they are described as [58]

(1) $$\mathrm{CBO}=\Delta E_{c}=\chi_{absorber}-\chi_{ETL}$$

(2) $$\mathrm{VBO}=\Delta E_{v}={\left(\chi +E_{g}\right)}_{HTL}-{\left(\chi +E_{g}\right)}_{absorber}$$

**Figure 4:**
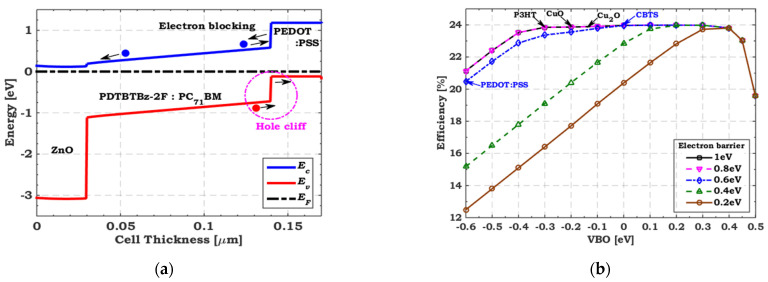
(**a**) Energy band profiles of tandem top cell with initial transport layers (ZnO and PEDOT:PSS) and (**b**) A theoretical study for conversion efficiency dependency on VBO of top HTL layer with different electron barriers.

**Figure 5:**
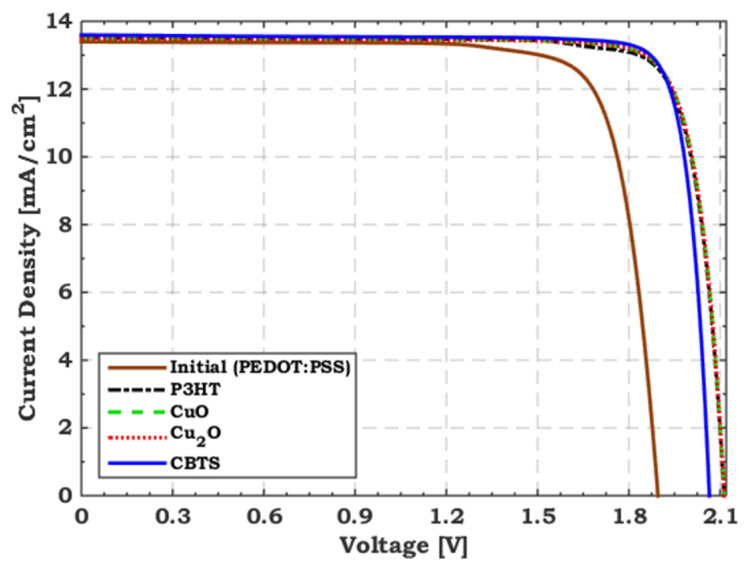
A comparative analysis between the illuminated *J–V* characteristics for the initial and TSCs utilizing P3HT, CuO, Cu_2_O and CBTS as top HTLs.

Initially, ZnO and PEDOT:PSS are utilized for the top cell as an ETL and HTL, respectively. The CBO for ZnO is –0.06 eV (almost flat band), and the VBO for PEDOT:PSS is −0.6 eV (large hole cliff), as depicted in Figure 4a. The recombination at the interface is well recognized to be critical due to the cliff-like band offset [59]. In thin-film solar cells, it is generally preferred that the bands be flat or have a slight spike-like band offset [60]. Therefore, we must substitute PEDOT:PSS with a suitable HTL to adjust the interface band alignment. As a theoretical study, VBO is varied from −0.6 to 0.5 eV with different electron barrier values. Figure 4b exhibits the tandem conversion efficiency with different electron barriers as a function of the VBO of the Polymer/PEDOT:PSS interface. As depicted in Figure 4b, the optimum range of VBO is from −0.3 to 0.3 eV with an electron barrier higher than 0.8 eV. To get the best tandem performance, we apply various materials such as copper barium thiostannate (CBTS), copper oxide (CuO), cuprous oxide (Cu_2_O), and poly−3-hexylthiophene (P3HT), which fulfill the optimum VBO and electron barrier values. Table 4 summarizes these materials’ main required parameters, indicating each material’s VBO and electron barrier values. In addition, Figure 5 compares the illuminated *J–V* characteristics regarding the initial and TSCs when employing P3HT, CuO, Cu_2_O, and CBTS as top HTLs. Their PV parameters are presented in Table 5. It is clear that when the top cell VBO is adequately designed, the tandem FF is enhanced and, thus, the overall performance is improved. The highest performance is achieved when CBTS is utilized as a top HTL, resulting in an improvement of 19% over the initial TSC.

**Figure 6:**
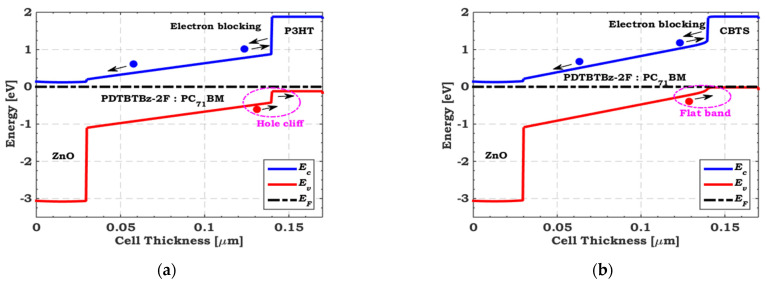
Energy band diagrams of (**a**) a cliff-like band occurs concerning P3HT/Absorber and (**b**) a flat band concerning CBTS/Absorber.

Figure 6 illustrates the energy band profiles of two different VBOs, which physically interpret the findings in Figure 5. First, the VBO for P3HT is –0.3 eV, which is indicated by the cliff-like band in Figure 6a. The second for CBTS, displayed in Figure 6b, has a flat band. The cliff-like band helps to extract photogenerated holes from the absorber film to the HTL, but it influences the activation energy required for carrier recombination. Thus, the principal recombination mechanism in the cell is recombination losses at the interfaces, where the activation energy (*E*_a_) is less than the absorber bandgap (*E*_g_), resulting in fill factor degradation [58,61]. Finally, as shown in Figure 6b, a flat band is created at the CBTS/Absorber interface. In this case, the carrier flow is not interrupted and *E*_a_ remains unaffected, leading to a higher FF value. Consequently, the flat band case is considered to be the most appropriate case. Therefore, with PCE = 24.32%, CBTS is the best choice for the top HTL.

**Table 4:** Basic parameters of various top HTL materials.

Parameters	Description	CBTS	P3HT	Cu_2_O	CuO
*E*_g_ (eV)	Energy gap	1.9	2	2.17	2.1
*χ* (eV)	Electron affinity	3.6	3.2	3.2	3.2
VBO (eV)	Valence band offset	0	−0.3	−0.13	−0.2
EB (eV)	Electron barrier	0.6	1	1	1
*ε_r_*	Relative permittivity	5.4	3	7.1	7.11
*μ_n_* (cm^2^/Vs)	Electron mobility	30	1 × 10^−4^	200	3.4
*μ_p_* (cm^2^/Vs)	Hole mobility	10	1 × 10^−3^	80	3.4
*N_c_* (cm^−3^)	Conduction band effective DOS	2.2 × 10^18^	1 × 10^21^	2.5×10^18^	2.2 × 10^18^
*N_v_* (cm^−3^)	Valence band effective DOS	1.8 × 10^19^	1 × 10^21^	1.8×10^19^	1.8 × 10^18^
*References*		[62]	[5]	[63]	[64]

**Table 5:** PV parameters for the initial and TSCs using P3HT, CuO, Cu_2_O and CBTS as top HTLs.

Top HTL	VBO	Electron Barrier (eV)	*V*_oc_ (V)	*J*_sc_ (mA/cm^2^)	FF (%)	PCE (%)
Initial (PEDOT:PSS)	−0.60	0.60	1.89	13.40	80.54	20.45
P3HT	−0.30	1	2.11	13.52	83.85	23.95
CuO	−0.20	1	2.11	13.51	84.38	24.10
Cu_2_O	−0.13	1	2.11	13.50	84.48	24.11
CBTS	0	0.60	2.06	13.60	86.67	24.32

### 3.2. Defect Density of the Front Polymer Absorber

**Figure 7:**
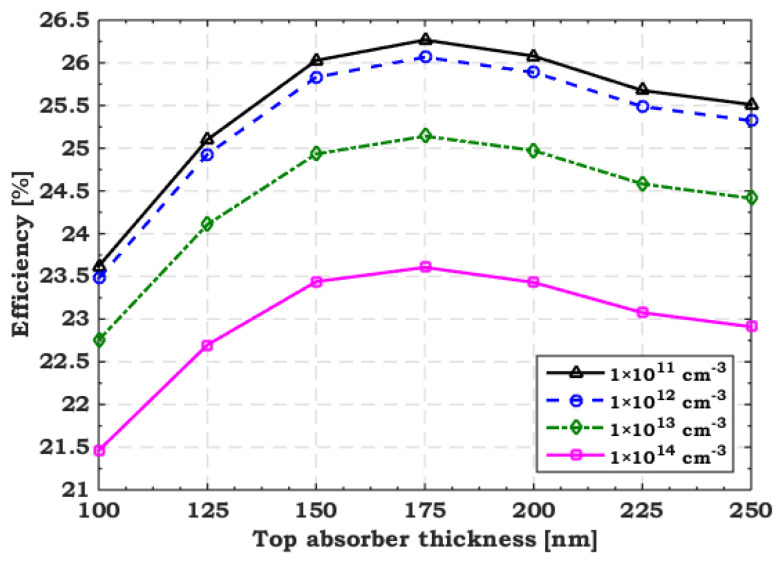
Tandem efficiency dependence on the variation of the thickness and defect density of the top absorber.

**Figure 8:**
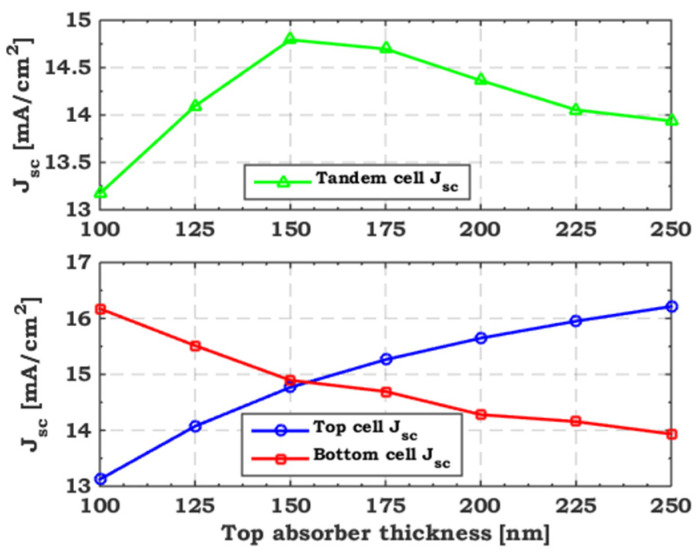
Variation of *J*_sc_ of the sub cells and that of the TSC with different top absorber thicknesses and a defect density of 1 × 10^11^ cm^−3^.

Figure 7 illustrates the variation in TSC efficiency versus the change in the top absorber thickness through different defect densities. We range the defect density from 1× 10^11^ to 1 × 10^14^ cm^−3^, while the thickness varies from 100 to 250 nm. As the figure concludes, the tandem efficiency degrades at a fixed thickness with increasing defect density due to increased carrier recombination (lower diffusion lengths). On the other hand, the tandem efficiency follows the same trend through different defect densities while changing the top absorber thickness. It steadily increases when the thickness does not exceed 175 nm and then gradually degrades when it surpasses this value. To interpret this finding physically, Figure 8 plots the variation in the *J*_sc_ of the two sub cells and that of the TSC with different top absorber thicknesses and a defect density of 1 × 10^11^ cm^−3^. As expected, increasing the top absorber thickness leads to more photon absorption and, thus, less transported light to the rear sub cell. Therefore, increasing the top absorber thickness increases the front cell current and reduces that of the rear cell. The smaller *J*_sc_ transporting through the two sub cells controls the *J*_sc_ of the 2T TSC. So, the tandem current beyond a 150 nm thickness behaves like that of the bottom cell, reducing efficiency. Therefore, the CMP condition must be fulfilled to diminish the decrease in tandem efficiency.

### 3.3. Thicknesses of the Absorber Layers

**Figure 9:**
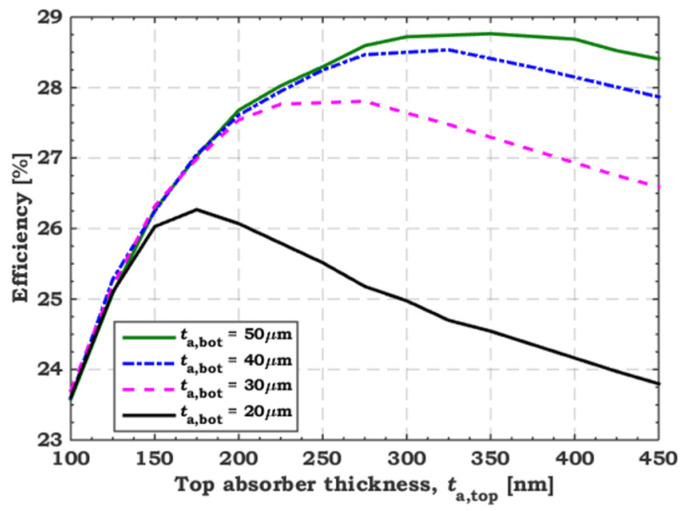
Tandem efficiency dependency on the thickness of the two absorber films.

Figure 9 signifies the dependency of the tandem efficiency on the thickness of both absorber layers. We varied the front polymer thickness (*t*_a,top_) from 100 to 450 nm, while the bottom absorber (*t*_a,bot_) was varied in the range (20–50 μm). As can be inferred from the figure, the PCE was unaffected by increasing *t*_a,bot_ from 30 to 50 μm and keeping *t*_a,top_ below 200 nm. As *t*_a,top_ falls lower than 150 nm, the PCE steadily reduces from 26.5% to 23.5%. In addition, changing *t*_a,bot_ from 30 to 50 μm does not affect PCE while keeping *t*_a,top_ constant below 150 nm. The best tandem performance is achieved with *t*_a,top_ = 250 nm and *t*_a,bot_ = 40 μm, providing the following PV parameters: *J*_sc_ = 16.18 mA/cm^2^, *V*_oc_ = 2.04 V, FF = 85.44%, and PCE = 28.22%.

### 3.4. Current Matching Point

**Figure 10:**
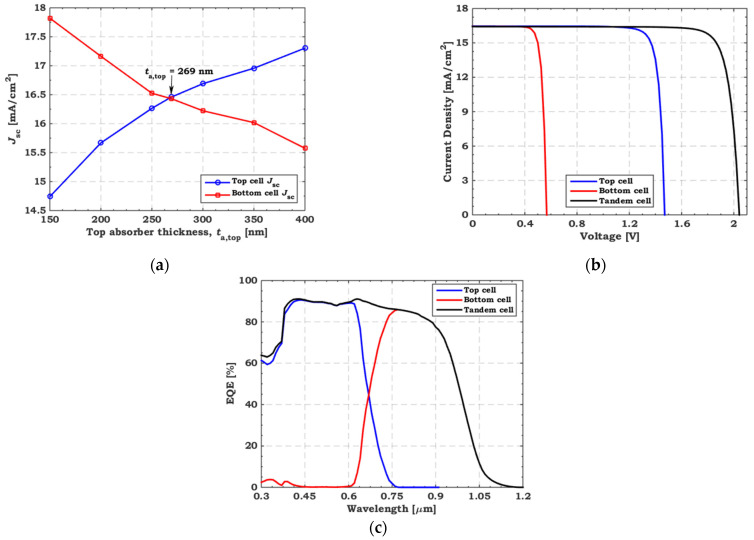
(**a**) *J*_sc_ dependence of the two sub cells on the top absorber thickness (*t*_a,top_), (**b**) illuminated *J–V* and (**c**) *EQE* spectra of the polymer/Si TSC and its sub cells under CMP.

In this sub section, we inspect the CMP by changing *t*_a,top_ from 150 to 400 nm while keeping *t*_a,bot_ fixed at 40 μm. Figure 10a depicts the *J*_sc_ dependence of the two sub cells on *t*_a,top_. As mentioned before, increasing the *t*_a,top_ leads to more photon absorption and, thus, less transferred light to the rear cell. Therefore, increasing the *t*_a,top_ increases the *J*_sc_ of the top cell and reduces that of the bottom cell, confirmed by the results in Figure 10. A CMP ensues at *J*_sc_ = 16.43 mA/cm^2^ and *t*_a,top_ = 269 nm. The TSC has been simulated by applying this condition, and Figure 10b shows the illuminated *J–V* characteristics for both the TSC and its sub cells. Their corresponding PV parameters are documented in Table 6. The maximum value of *J*_sc_ is 16.43 mA/cm^2^ with *V*_oc_ = 2.04 V and PCE = 28.41% for the TSC. *V*_oc_ = 2.04 V equals the sum of the top cell (1.47 V) and bottom cell (0.57 V), demonstrating the efficient operation of the recombination junction. Moreover, the *EQE* of the TSC and its sub cells at CMP is exhibited in Figure 10c. The *EQE* of the back c-Si sub cell exceeds 85% at a wavelength of around 800 nm.

Finally, Table 7 presents a state-of-art comparison between our 2T monolithic polymer/Si TSC and some multi-junction TSCs reported in the literature. The table demonstrates that some tandems are derived from experimental investigations, while others are based on numerical calculations. Experimentally, until now, the efficiency of a 2T polymer/Si TSC has been limited to 9%. Furthermore, while lead-based perovskite TSCs achieve higher efficiencies than our suggested thin-film TSC, toxicity is a significant issue that restricts their use. Moreover, our simulated polymer/Si TSC exhibits favorable characteristics with a high *V*_oc_ and PCE.

## 4. Conclusions

In this work, we presented a proposed 2T monolithic polymer/Si TSC. In the suggested design, the front cell blends two organic materials (PDTBTBz-2F as the polymer donor and PC_71_BM as the fullerene acceptor), while thin c-Si is invoked as the rear sub cell. The separate sub cells are calibrated against experimental investigations, providing a PCE of 9.88% and 14.26% for the front and rear cells, respectively. Then, we investigated the VBO impact of the front cell on the TSC performance. Furthermore, we inspected the influences of the top polymer defect density as well as both absorber thicknesses for better tandem performance. After optimizing the TSC and at the designed CMP, the *J*_sc_ and PCE of the tandem cell are enhanced to 16.43 mA/cm^2^ and 28.41%, respectively. These findings suggest that the proposed design can pave the way for flexible, environmentally friendly, and high-efficiency tandem cells.

## Figures and Tables

**Table 6:** PV parameters of polymer/Si TSC at CMP.

PV Parameters	*V*_oc_ (V)	*J*_sc_ (mA/cm^2^)	FF (%)	PCE (%)
Top cell	1.47	16.43	86.26	20.87
Bottom cell	0.57	16.43	80.75	7.54
Tandem cell	2.04	16.43	84.81	28.41

**Table 7:** A state-of-art comparison between PV metrics of polymer/Si TSC and some TSCs reported in the literature.

Top Cell	Bottom Cell	Method	*J*_sc_ (mA/cm^2^)	*V*_oc_ (V)	FF (%)	PCE (%)	REF
polymer: organic PBDB-T-2F:Y6	Si	Exp.	15.81	1.08	55.57	8.32	[65]
polymer: organic PBDB-T:ITIC	Si	Exp.	-	-	-	15.25 *	[65]
Lead-based Perovskite	organic PBDB-T-2F:Y6:P_71_CBM	Exp.	14.56	2.13	75.60	23.40	[9]
Lead-based Perovskite	CIGS	Exp.	19.24	1.768	72.90	24.20	[9]
Lead-based Perovskite	Si	Exp.	20.24	1.979	81.20	32.50	[30]
Sb_2_S_3_	Si	Sim.	18.04	1.64	82.41	24.34	[37]
Lead-free Perovskite	Si	Sim.	16.01	1.76	86.70	24.40	[31]
Lead-based Perovskite	CIGS	Sim.	20.49	1.81	81.80	30.50	[33]
polymer: fullerene (PDTBTBz-2F: PC_71_BM)	Si	Sim.	16.43	2.04	84.81	28.41	This work

Sim. = simulation and Exp. = experiment, *: it is a 4T tandem cell.

## Data Availability

No new data were created or analyzed in this study. Data sharing does not apply to this article.
